# Feeding of Undocked Pigs: Effects of Feed Components and Feed Composition

**DOI:** 10.3390/ani16081174

**Published:** 2026-04-11

**Authors:** Frederik Loewenstein, Sebastian Mascher, Tanja Frey, Mirjam Lechner

**Affiliations:** 1 LSZ Boxberg, Seehöfer Str. 50, 97944 Boxberg, Germany; 2 Tierärzte Team Tiefenbach, Steigäckerweg 10, 74564 Crailsheim, Germany; s.mascher@tierarzt-crailsheim.de; 3 Pig Health Service Baden-Württemberg, Schaflandstr. 3/3, 70736 Fellbach, Germany; t.frey@tsk-bw-tgd.de; 4 Independent Researcher, Am Wasen 20, 91567 Herrieden, Germany; mirjam.lechner@web.de

**Keywords:** undocked pigs, tail biting, SINS, gut health, feeding, feed components

## Abstract

Tail biting is a widespread problem in pig production worldwide and a major cause of reduced welfare in pigs. To prevent this harmful behavior, it is a common practice to optimize husbandry and enrich the environment with organic materials. Fiber-rich materials such as straw, hay, or alfalfa reduce tail biting. These observations were also made for swine inflammation and necrosis syndrome (SINS), which causes acral necrosis and increase the risk for tail biting. A supplementation of the diet with straw and hay significantly reduced systemic inflammation in pigs. Organic materials and feed components play an important role in preventing tail biting and SINS. Gut health in particular is influenced by diet and its components. Different effects of nutritional ingredients could disrupt the balance of the gut microbiota, damage the intestinal barrier and results in leaky gut and systemic inflammation. This inflammatory response can induce SINS and behavioral changes. The aim of this literature review is to summarize current knowledge on the effects of feeding and gut health on the occurrence of tail biting and SINS in pigs and to derive possible measures for prevention.

## Simple Summary

Tail biting is a common animal welfare problem in pig production worldwide. Farmers try to prevent tail biting by improving housing conditions and providing organic enrichment materials. Fiber-rich materials such as straw, hay, or alfalfa are known to reduce tail biting. Similar effects have also been observed for swine inflammation and necrosis syndrome (SINS), a disease associated with systemic inflammation and tissue damage in pigs. Nutrition plays an important role for pig health. In particular, gut health is strongly influenced by diet and changes in the gut microbiota. If the intestinal barrier is damaged, harmful substances can pass into the bloodstream and cause systemic inflammation. This so-called “leaky gut” may contribute to the development of SINS and to behavioral changes such as tail biting. This literature review summarizes current knowledge on how feeding strategies and gut health are linked to tail biting and SINS in pigs. Based on existing studies, possible nutritional and management measures to prevent these problems are discussed.

## 1. Introduction

Tail biting in pigs is a damaging behavior that causes injuries and can result in infections in bitten pigs. It is a common problem in modern pig production that reduces animal welfare and health [1,2]. Different animal-, husbandry- and management-related risk factors have been identified in association with tail biting [3,4]. Aggressive behavior, lack and quality of enrichment material, air quality, feeding space, water supply and overcrowding are identified as important factors [4,5,6,7]. At the same time, systemic inflammation is observed more frequently in connection with tail biting. The newly established swine inflammation and necrosis syndrome (SINS) is often identified as the cause of systemic inflammation [8]. The pigs show clinical signs of inflammation and necrosis in acral areas. Various parts of the body are affected by inflammatory lesions and necrosis, such as tail base, tail tip, ears, coronary bands, heels, soles, claw walls, teats, navel and face. Necrotic tail lesions caused by SINS are a risk factor for tail biting in pigs [8]. The severity of clinical signs related of SINS, similar to tail biting, depends on factors such as feeding, water supply, and various husbandry factors [8,9,10,11,12,13].

To prevent tail biting in pigs, manipulable material is used to enrich the holding systems [4,14]. The preventing effects depend on the quality of the material. Non-organic enrichment material such as metal or plastic objects has no significant effect on tail biting. In different studies roughage, compost, hessian sacks and fresh wood effectively reduce tail damage [15]. Straw as bedding and environmental enrichment material reduce tail biting in growing and finishing pigs [16,17]. In a study with undocked pigs, the prevalence of tail biting was reduced by 50% with straw. The tail length losses were less than 5 mm and could only be recorded by precise clinical examination [18]. Hay, haylage, alfalfa hay, grass silage and corn silage also have positive effects on the occurrence of tail biting [19,20,21]. Similar effects of supplementary straw and hay feeding were observed in the severity of SINS. A daily feeding of straw to sows and hay during the rearing and fattening period meant that SINS lesions were reduced in all production stages. Coprostasis in sows especially had a significant impact on the severity of SINS in suckling piglets and weaners [9]. Constipation (coprostasis) is a leading symptom of postpartum dysgalactia syndrome (PPDS) in sows. Crude fiber and water supply are identified as important risk factors for coprostasis [22]. Similar to PPDS in sows, coprostasis plays a significant role in the pathogenesis of SINS. Fiber content in feed and roughage are crucial factors in preventing coprostasis and avoiding SINS [8]. In addition, dietary fiber can regulate the gut microbiota and microbial metabolites (e.g., short-chain fatty acids, SCFA). The addition of different fiber sources to the diet significantly reduced gut and systemic inflammation in sows and piglets. Supplementation with alfalfa meal significantly increased the relative abundance of anti-inflammatory bacteria (e.g., *Prevotellaceae*, *Lachnospiraceae*) and decreased pro-inflammatory bacteria (e.g., *Desulfovibrio* spp., *Helicobacter* spp.) [23].

These observations suggest that gut health influences the incidence of systemic inflammation and affects the behavior of pigs. The aim of this literature review is to summarize current knowledge on the effects of feeding and gut health on the occurrence of SINS and tail biting in pigs in order to identify possible measures for the future. Initially, the background of tail biting in pigs and the effects of feeding on the occurrence of this behavioral disorder are described. Gut health plays a crucial role in this context. Damaging influences on intestinal integrity are first summarized in general and then related to aggressive behavior, systemic inflammation and SINS. Based on the pathogenesis of tail biting and SINS, as well as the effects on gut health, the hypothesis of leaky gut-associated systemic inflammation as a common cause of SINS and tail biting is derived. In order to provide a deeper understanding of the effects of feeding on gut health, the influences of selected feed components are examined in detail and their damaging or beneficial effects on intestinal integrity are highlighted. On this basis, feeding strategies and measures for optimizing and preventing both clinical conditions are deduced.

## 2. Tail Biting in Pigs

Tail biting is a major welfare and economic problem in modern pig production worldwide. Economic losses caused by tail biting result in costs of up to €7.63 per piglet during rearing [24] and up to €18.96 per pig during fattening [24,25]. The increased production costs per pig are the result of lower daily weight gain, increased medical treatment, increased labor, more enrichment materials, partial losses, and complete condemnation at slaughter [26].

Tail biting is an abnormal behavior, characterized by the wound-inducing oral manipulation of one pig’s tail by another pig. For over 70 years, preventive tail docking has been a common practice to prevent harmful behavior in pigs [19,27]. In Europe, routine tail docking has been prohibited by law since 1994. Tail docking is only permitted in cases of tail biting. A survey of 24 European countries revealed an average proportion of 77% of pigs with docked tails. In Finland, Norway, Sweden, and Switzerland, only 1.5%, 0%, 0%, and 2.5% are tail docked, respectively [28]. The length of the docked tail influences tail-directed behavior and the occurrence of tail biting [29]. How tail docking prevents tail biting has not yet been conclusively determined. Paoli et al. [30] suggest that longer tails are easier for pigs to bite with their cheek teeth. This assumption has not yet been confirmed in other studies. However, tail biting occurs more frequently in undocked pigs [31].

There are different types of tail biting in pigs. Taylor et al. [1] divide tail biting in pigs into the following three different types: two-stage tail biting, sudden-force tail biting and obsessive tail biting. Two-stage tail biting occurs most frequently in pigs. This type is characterized by a pre-damaging stage and a damaging stage. First, a pig manipulates the tail of another pig with its mouth. This is tolerated by the affected pig (pre-damaging stage). If an injury is caused to the tail, tail biting occurs (damaging stage) [1]. The tail-in-mouth behavior during the pre-damaging stage is attributed to the normal behavior of pigs [1,28]. The reason for the acceptance of pigs that have been manipulated at the tail is not yet known. Tail necrosis that is not caused by bite injuries is described as a risk factor for tail biting [11,12]. The second type of tail biting is triggered by a lack of particular resources and is characterized by sudden and forceful biting behavior. The third type of tail biting is defined by aggressive, abnormal behavior in individual pigs. The origin of this obsessive tail biting is unknown [1]. The various forms of tail biting suggest that close observation of the pigs is necessary for the early detection of risks and signs of tail biting.

Risk factors include, for example, early bite injuries. Pre-weaning pigs that had already been bitten were significantly more likely to become victims of tail biting again in all production stages. For the prevention of tail biting, the authors suggest observing behaviors such as chewing or manipulating objects in the pens to predict tail biting [32]. In contrast, another study found no change in activity, feeding, exploration or tail-directed behavior prior to tail biting outbreak. However, a significant correlation was found between tail posture and tail biting. In pens with tail biting, 33.2% of the pigs showed hanging tails one day before biting behavior started [33]. This is consistent with observations in other studies [34,35,36,37]. In 1943, the first description of the curly tail as an indicator of animal welfare was published [38]. A hanging tail in pigs has been attributed to relaxation or paralysis of the tail muscles during fever, digestive disorders, or poor feed quality; historically, a curled tail indicated health, whereas febrile or indigestion-related conditions caused the tail to hang limp [38]. Comparable mechanisms are known in humans, where systemic inflammation and infection impair skeletal muscle by proteolytic degradation of contractile and cytoskeletal proteins, leading to functional weakness proteolytic processes cleave important components of the skeletal muscle contractile apparatus and cytoskeleton including actin, actinin, spectrin, myosin and talin [39]. This concept aligns with findings in dogs, where limber tail represents an acute inflammatory myopathy, with coccygeal muscle degeneration and necrosis producing a paralyzed, hanging tail [40]. In pigs, systemic inflammatory processes such as SINS induce local edema, vascular permeability, and immune-cell infiltration at the tail base, indicating subclinical inflammation with potential neuromuscular impact [41]. Additionally, widespread inflammatory reactions and neural regression occur even in tails deemed clinically normal [42]. These findings support the hypothesis that fever, systemic inflammation, or chronic inflammatory syndromes may lead to tail muscle dysfunction and a hanging tail in pigs, warranting targeted investigation. However, the pathological causes of hanging tails in pigs must be viewed separately from natural tail posture. The tail posture also plays a decisive role in pig communication, where a hanging tail is associated with a positive emotional state [43]. In conclusion, this means that the condition of the hanging tail must be considered in relation to the health status of the pig. At the same time, it can be suspected that a hanging tail is more susceptible to being manipulated by other pigs with their mouth.

Based on tail-in-mouth behavior, manipulable enrichment materials are offered to prevent harmful behavior [14,44]. Enrichment material for pigs should be investigable, manipulable, chewable and edible. To maintain attractiveness of the material, a regular change and the simultaneous use of different enrichment is essential [45]. The effect of enrichment material is measured by the reduction in harmful behavior and behavioral disorders [4,15,46,47,48]. This has been verified in numerous studies on different materials (reviewed in [15]) and formed the European legislation [3,4,49]. However, dietary and metabolic effects are also hypothesized from organic enrichment material [50]. This may result in potential preventive strategies for ration composition.

### Feeding as Preventing Factor

Feeding is assessed as a risk factor for tail biting in pigs based on the animal-feeding space ratio. Insufficient feeding space promotes aggressive behavior. In a study of docked rearing pigs, no lesions on tails and ears were found in the ratio of 3.75 pigs per feeder. With an increased animal-to-feeder ratio of 7.50, significantly higher prevalences of tail and ear lesions were observed at 11.9% and 5.7% respectively [51]. The width of the feeders also has a significant impact on the prevalence of tail lesions in docked pigs. Fewer feeding spaces per pig result in a higher incidence of lesions [52]. The effects of feed ratio on tail biting have been neglected and rarely investigated [50]. In their study, Kallio et al. [6] found no effects of feeding on tail biting in pigs. However, data on feeding from farms were collected and evaluated as part of a survey. Dietary effects of the rations were not investigated in detail [6].

In contrast, in a study from Sweden, a ration containing levels of amino acids, minerals, and vitamins that were above the national standard was fed to fattening pigs [53]. The amino acid content (lysine, methionine, threonine, and tryptophan) was increased by 10% above the national standard. Calcium, phosphorus, sodium chlorine and magnesium levels were increased by 20% above the standard. Vitamins A, B_1_, B_2_, B_3_, B_5_, B_6_ and B_12_ were increased by 10–20%, vitamin D was increased by 40% and vitamin E was increased by 80% above the standard. In addition, increased fiber was fed with pellets and haylage. The authors conclude a significant reduction in tail biting and suspect additional effects from the supplementation of haylage on the incidence of tail biting [53]. Similar effects are described for reduced crude protein content. In a study on docked pigs, a 20% reduction in crude protein compared to the standard diet significantly increased the prevalence of tail biting up to 25% during rearing to fattening [54]. In order to evaluate the influence of feeding on the prevalence of undocked pigs, ingredients and nutritional components in the ration are necessary. However, complete diet compositions are only available in a few studies. Some of these rations from various studies are summarized in Table 1.

The cited studies examined the effects of fiber [56,57,59,60,61], protein [55], amino acids [56], and enrichment material [57,58]. The effects of crude fiber in the diet of undocked pigs show different results. In a study with increased fiber content of the ration and one organic or non-organic toy for weaners and finishers decreased to 5.6% and 11.6% respectively (Table 1). A reduction in tail biting could not be obtained [57]. In comparison, a study by Roux et al. [56] increased the proportion of fermentable fiber to 6% and used content of 0.19% digestible tryptophan in weaners and finishers. A significant increase in intact tails was observed in both age groups [56]. However, combination effects or solitary effects of tryptophan cannot be ruled out. The use of straw pellets had no effect on the occurrence of tail biting in weaners, while a trend could be observed in finishers, in the authors’ opinion [59]. A combination of straw pellets and alfalfa increased the proportion of intact tails in weaners. In finishers, the use of chopped hay had no effect on the prevalence of tail biting [61]. The results for different roughage types indicate that the effects of fiber content depend on the type of roughage and possible combination effects when used simultaneously. Further studies on undocked pigs are necessary to determine the causal effects of roughage.

The effects of protein content on tail biting in undocked pigs were investigated in a study by Roch et al. [55]. They observed a prevalence of 14.7% in tail biting with a protein reduction of 20% in the diet (Table 1). A reduction in the protein content of the diet leads to an insufficient supply of essential amino acids like tryptophan [54]. Differential effects of additional tryptophan substitution on tail biting were observed. In a study with docked pigs, an increase in tryptophan content of up to 250% compared to the standard diet led to an increase in circulating tryptophan in plasma. However, no effects on tail biting were observed [62]. In the study of Roux et al. [56] with undocked pigs, the tryptophan content of the standard diet (0.19% tryptophan) was reduced (0.15% tryptophan). Tail biting was observed in up to 70% of pigs with reduced tryptophan content in the diet [56]. Similar effects on aggressive behavior in pigs have been described with a reduced lysine content (80% lysine of standard diet) in the diet. The authors suggest that nutritional deficiency in lysine and an impaired immune response increase ear biting [63]. The results of the cited studies on undocked pigs imply that protein and amino acid levels in the diet can have a significant impact. At the same time, very high levels of tryptophan do not increase positive effects on prevalence of tail biting. On the other hand, too low tryptophan content is a possible risk factor for tail biting.

The effects of various feed components and ingredients on tail biting have been described in undocked pigs and discussed based on the available literature. The exact mechanisms by which fiber, protein, and amino acid content influence the occurrence of tail biting are unclear. One approach to determining possible impacts on pigs is to focus on the effects on gut health.

## 3. Effects on Gut Health of Pigs

The gut health of pigs is becoming increasingly important [64,65,66]. In general, gut health is defined as the absence, prevention or avoidance of intestinal diseases. More broadly, the definition can include positive aspects such as digestion and absorption of feed, intact intestinal barrier, normal and stable gut microbiota, effective immune status and a functional enteric nervous system [64,65]. The pig’s intestine is the body’s largest immune organ. More than 70% of the body’s immune cells are located in the intestine [67]. There are intense interactions between the intestinal microbiota and the intestinal immune system. The secretion of various immune factors regulates the composition and homeostasis of intestinal microorganisms [68], while intestinal microorganisms stimulate the differentiation of immune cells (such as T-cells) via specific components or metabolites. An intact gut is therefore essential for the welfare and production efficiency of pigs of all ages [68,69].

The gut microbiota contains commensal, symbiotic and pathogenic microorganisms, including bacteria, viruses, parasites, fungi, archeae and protists. A healthy gut microbiota is characterized by diversity, stability, resistance and resilience [70]. The composition of the microbiota depends on the genetics of the host, the environment and the feeding [71,72,73]. *Lactobacillus* spp. and *Clostridium* spp. are mainly present in the small intestine, while *Prevotella* spp. dominate in the large intestine [74,75]. A stable gut microbiota is an important component in maintaining gut health and stabilizing the intestinal barrier [76].

**Figure 1:**
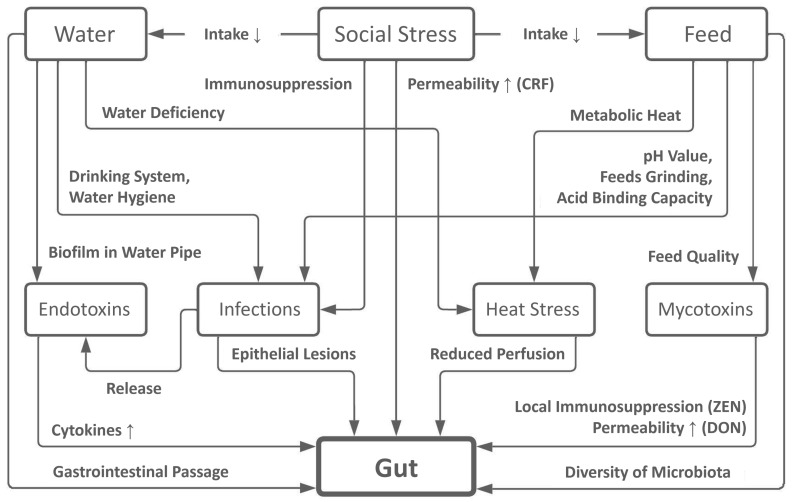
Influences on the gut health of pigs. CRF, corticotropin-releasing factor; DON, deoxynivalenol; ZEN, zearalenone; ↑, increase; ↓, decrease.

The integrity and permeability of the intestinal barrier depends on the intrauterine development and the age of the pigs [77,78,79]. Damage of the intestinal barrier can be caused by ration composition [23,80], water deficiency [81] and quality [82], heat stress [83], infections [84], mycotoxins [85] and social stress [86]. The effects on gut health and the intestinal barrier are summarized in Figure 1.

Social stress caused by changing pens, regrouping, rank fights, or overcrowding results in reduced water and feed intake. Immunosuppressive effects increase the risk of infection. In addition, stress leads to increased permeability of the intestinal wall due to the corticotropin-releasing factor [86].

Water intake affects the development of heat stress, infections, endotoxin and directly impacts gut health. Water deficiency reduces body’s thermoregulatory capacity and leads to reduced perfusion to the intestines. Direct damage to the intestinal barrier increases permeability [83]. The cleanliness and type of drinking systems, as well as water hygiene, can promote infections with intestinal pathogens [81,82]. In addition, biofilms in water pipes are a risk factor for increased endotoxin contamination in water. Endotoxins, also released by infectious agents in the gut, stimulate the immune system and trigger an inflammatory response with increased cytokine activity in the gut. Insufficient water intake also has a direct effect on the gastrointestinal passage of feces [81]. Coprostasis prolongs the passage time and promotes microbial proliferation [9,22].

Feed intake also affects the development of heat stress, mycotoxin intoxication, infections and the diversity of the microbiota, and thus indirectly affects gut health. The resulting digestive heat promotes the development of heat stress when thermoregulatory mechanisms are insufficient. Various feed characteristics such as high pH, fine grinding, and high acid-binding capacity predispose pigs to intestinal infections [23,80]. Intestinal pathogens damage the intestinal wall and cause epithelial lesions [84]. Mycotoxins in feed directly affect intestinal health. Deoxynivalenol (DON) damages the intestinal wall and causes increased permeability, while zearalenone (ZEN) can trigger local immunosuppression in the gut [85].

A combination of these factors leads to the development of leaky gut, a condition that leads to increased permeability of the intestinal wall. Damage to enterocytes and dysfunctional tight junctions cause unregulated transcellular and paracellular transport of various nutrients and toxins, as well as the passage of microbial organisms can trigger systemic inflammatory response [87,88]. Due to leaky gut, effects on the brain caused by increased circulating microbial metabolites in the bloodstream have also been described [89]. This suggests that various effects on gut health and the resulting leaky gut may influence the behavior and promote systemic inflammation in pigs.

### 3.1. Influences on Harmful Behavior

Behavior is influenced by the microbiota-gut–brain axis (MGBA). Bidirectional communication between microbiota and brain occurs via various pathways. The vagus nerve, the immune system, neuroendocrine pathways and bacteria-derived metabolites are involved [90,91,92]. Commensal bacteria interact with enteroendocrine cells in the intestinal epithelium via metabolites (like SCFA) and regulate the secretion of various gut peptides [91]. Microbial-derived metabolites and neurotransmitters are also absorbed out of the intestinal lumen and enter the bloodstream. SCFAs like acetate, butyrate and propionate are the dominant metabolites. Gut microbiota-associated synthesized neurotransmitters include serotonin, melatonin, gamma-aminobutyric acid, catecholamine, acetylcholine, and histamine [91,93]. Paracellular transfer of metabolites can occur in the case of leaky gut. Increased amounts of circulating metabolites and neurotransmitters can have regulatory effects in the brain [89].

In pigs, correlations between microbiota and various effects on behaviors have been described, for example, tail biting and anxiety [94]. A relatively high abundance of the families *Lachnospiraceae*, *Ruminococcaceae*, and *Clostridiales* of the phylum Firmicutes was associated with tail biting in pigs [95]. *Clostridiales* and in particular *Ruminococcaceae* and *Lachnospiraceae* affect the 5-hydroxytryptamine (5-HT or serotonin) biosynthesis in the gut [96]. Verbeek et al. [95] suggest a possible correlation between microbial influences on serotonin metabolism and the occurrence of tail biting. This speculation is supported by increased serotonin metabolism in the prefrontal cortex of tail biters [97]. Comparable serotonin-associated behavioral changes have also been suspected in humans. However, the serotonin theory of depression in humans is now considered obsolete. Research areas on serotonin have not provided consistent evidence of a link between low serotonin levels in the brain and depression [98].

Of the body’s total 5-HT content, 95% is located in the gut and 5% in the brain. Enterochromaffin cells synthesize and store 90% of the 5-HT content of the gut and a further 10% is found in enteric neurons [99]. The gut microbiota plays a crucial role in the regulation of the serotinergic system by altering the expression of 5-HT-related genes [100]. For example, the intestinal microbiota influences the transcription of enzyme tryptophan hydroxylase 1 (Tph 1) and increases 5-HT biosynthesis [101]. This open up possibilities to directly modify the MGBA via the diet and indirectly influence behavior and improve pig welfare [102]. Kobek-Kjeldager et al. [103] hypothesize that a sufficient supply of amino acids, including tryptophan, and higher dietary fiber levels in the feed promote a beneficial gut microbiota and can reduce tail biting in pigs. The effects of low tryptophan levels and various fiber content on the prevalence of tail biting have already been observed (Table 1) [56]. Whether this behavior is due to beneficial effects on the microbiota has not yet been clarified and must be verified in further studies.

### 3.2. Influences on Systemic Inflammation

Systemic inflammation can be caused by deficiencies in intestinal integrity. Disruption of the intestinal barrier leads to a leaky gut and results in the release of bacterial metabolites, endotoxins, such as lipopolysaccharides (LPSs) [104], and bacterial translocation [105] into the bloodstream. This condition is mainly caused by bacterial infections, oxidative stress, diet and dysbiosis [104]. Tryptophan metabolism also plays a crucial role in systemic inflammation in pigs and other vertebrae [106]. There are the following two tryptophan metabolic pathways: the kynurenine pathway and the serotonin pathway. Both metabolic pathways are essential for maintaining a healthy homoeostasis [107]. In an in vitro experiment with a rat adrenal pheochromocytoma (PC12) cell line, it was observed that increased expression of key genes of one metabolic pathway resulted in reduced expression of genes of the other [108]. The kynurenine pathway is the main metabolic pathway. Around 90% of dietary tryptophan is converted into kynurenine (KYN) and its derivatives. The kynurenine pathway is catalyzed by the enzymes tryptophan 2,3-dioxygenase (TDO) and indoleamine 2,3-dioxygenase (IDO) [109]. TDO catalyzes the basic tryptophan metabolism in the liver. IDO is mainly found in active immune cells and is induced by pro-inflammatory cytokines such as interferon-gamma (IFN-γ), interleukin-1 (IL-1), IL-6 and tumor necrosis-factor-alpha (TNF-α) [110]. Endotoxins like LPS cause increased IDO-induced activation of the kynurenine pathway [111]. KYN is an intermediate metabolite which can passage the blood–brain barrier and can be metabolized into various neuroactive products such as quinolinic acid (QUIN), kynurenic acid (KYNA) and 3-hydroxykynurenine (3-HK). QUIN and 3-HK cause neuronal inflammation, while KYNA has neuroprotective, anti-inflammatory and anti-depressant effects [112]. In pigs, KYNA, QUIN and 3-HK are associated with sickness behavior. Specifically, in the context of infection and inflammation, cytokines are related to increased activation of the kynurenine pathway. Neurotoxic effects of QUIN and 3-HK are possibly associated with tail biting [112].

#### Swine Inflammation and Necrosis Syndrome (SINS)

Systemic inflammation as a cause of disease syndromes such as PPDS has already been well described [22,113]. In the pathogenesis of SINS, leaky gut plays a crucial role (Figure 1) [8,9]. Clinical inflammation and necrosis affect the base of the tail, tail tip, ears, coronary bands, heels, soles, claw walls, teats, navel, face and vulva in suckling piglets, weaners and fattening pigs [8,9,41,114,115,116,117]. Effects on sows and boars as well as parental effects on their offspring have also been demonstrated [9,118,119,120,121,122,123]. SINS is an endogenous, systemic inflammation induced by endotoxins such as LPS and microbial-associated molecular patterns (MAMPs) from intestinal bacteria [8,124,125]. LPS and MAMPs translocated from the gut to the liver via the portal vein and were recognized by Toll-like receptor 4 (TLR-4) of macrophages in the liver [124]. Inflammation of the liver reduces the detoxification capacity. Endotoxins enter the circulation and interact with neutrophils, macrophages and platelets. This leads to the secretion of pro-inflammatory mediators such as TNF-α, IL-1β, IL-6 and IL-8 [8]. Activation of the endothelium induces adhesion molecules with increased docking of monocytes and granulocytes and their infiltration into the tissue. These changes result in increased blood coagulation and thrombosis of capillary blood vessels [8,118]. Based on the pathogenesis of SINS, avoiding leaky gut is a preventive measure. In previous studies, the prevalence of SINS in suckling piglets, rearing pigs, and fattening pigs was significantly reduced by supplementing hay [9]. In lactating sows, supplementing straw reduced coprostasis. Lower prevalence of coprostasis in sows was correlated with lower SINS severity in piglets [9,10,11]. Further studies are needed to determine the effects of feeding on SINS and possible feeding strategies.

Effects of SINS on the behavior of pigs are also observed. In addition to typical sickness behavior, a connection with tail biting is also suspected. Due to the itching at the tip of the tail, tail-in-mouth behavior by pen mates is accepted. As a result, the risk of two-stage tail biting increases [8]. In SINS-affected suckling piglets the expression of inflammation-associated genes was significantly increased compared to low affected suckling piglets. For example, TDO genes (fold change FC = 1.88; *p* = 0.03) were significantly more highly expressed [124]. As described above, the kynurenine pathway plays a key role in tryptophan metabolism in systemic inflammation [106,107,108,109]. Especially, endotoxins increased the IDO-induced activation of the kynurenine pathway [110].

### 3.3. Leaky Gut as a Cause of Tail Biting and SINS

**Figure 2:**
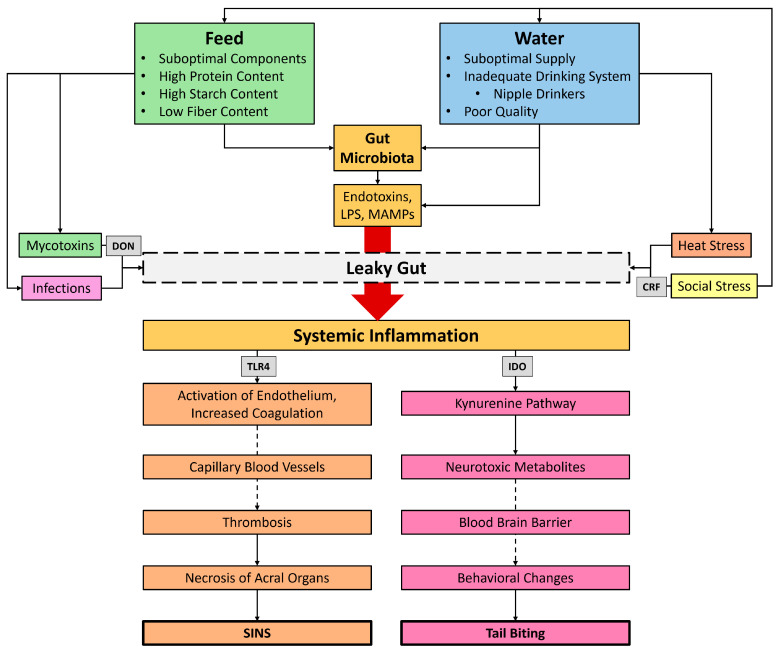
Hypothesis of leaky gut-associated systemic inflammation as a cause of swine inflammation and necrosis syndrome (SINS) and tail biting behavior in pigs. Increased transfer of endotoxins through a damaged gut barrier (leaky gut) leads to a systemic inflammatory response. On the one hand, this results in TLR4-mediated activation of the endothelium with increased coagulation (dashed arrows), thrombosis, and necrosis of acral organs. On the other hand, there is increased induction of IDO by pro-inflammatory cytokines and increased IDO-mediated activation of the kynurenine pathway by endotoxins, leading to increased formation of neurotoxic metabolites. Once the blood–brain barrier is crossed (dashed arrows), behavioral changes such as tail biting can be triggered. CRF, corticotropin-releasing factor; DON, deoxynivalenol; IDO, indoleamine 2,3-dioxygenase; LPS, lipopolysaccharide; MAMPs, microbial associated molecular patterns; TLR4, Toll-like receptor 4.

Gut health is influenced by various factors (Figure 1). Systemic inflammation triggered by a leaky gut can cause SINS [8,9,41,115,116,117,118] and, via the gut–brain axis, behavioral changes [99,100,101,102,103,104,105,106,107,108,109] in pigs. Based on the current literature, we hypothesize that the occurrence of a leaky gut and the associated systemic inflammation may be a cause of both SINS and tail biting in pigs (Figure 2).

According to our hypothesis, feeding strategies are necessary to prevent the development of leaky gut and thus tail biting and SINS. A better understanding of the impact of diets requires consideration of the effects of individual feed components on gut health.

## 4. Properties of Different Main Feed Components and Effects on Gut Health

As outlined in the previous sections, leaky gut could be a trigger of tail biting and SINS in pigs. This section reviews the effects of different feed components on gut health and the intestinal barrier integrity.

Functionality and health of the gut depend on diet, effective structure and function of the intestinal barrier, host interaction with the intestinal microbiota, effective digestion and absorption of feed and effective immune status [126,127,128]. In particular, feeding at suckling piglet age [129] and around the time of weaning has a major impact on gut health up to fattening age [128,130,131,132,133]. This is consistent with observations on the occurrence of tail biting and SINS. A high risk for tail biting is observed in pigs post-weaning and during the rearing phase [134,135], while SINS can be observed at all ages and to varying degrees [8,9,12]. This suggests that the influence of feeding on gut health is an important factor in the development of both clinical conditions.

In general, plant-based diets are primarily used in the feeding of pigs. Various cereals and legumes such as wheat, barley, oats, maize and soya are fed as main components [136]. Table 2 summarizes the effects of different main feed components on gut health. When these effects are considered in relation to the development of a leaky gut, the beneficial and harmful properties of the feed components can be highlighted. In general, wheat increases permeability of the intestinal barrier and has pro-inflammatory properties in the intestine [137,138,139]. However, the effects of the gluten fraction, lectin, and amylase-trypsin inhibitors are based on studies in humans. In contrast, the cereal components barley and oats have beneficial effects on intestinal health (Table 2). Barley influences the gut microbiota [140], strengthens local immune defense in the gut [141], and increases SCFA production [142]. Oats have similar beneficial effects on the gut microbiota and anti-inflammatory influences in the caecum and colon [143]. Maize has a positive effect on the gut microbiota and increases the production of various SCFAs [144,145,146]. Soybean meal can, depending on how it is processed, potentially disrupt intestinal integrity via β-Conglycinin and Glycinin and have a negative effect on intestinal microbiota [147,148,149]. However, isoflavones strengthen the intestinal barrier [150]. In summary, the feed components have various properties that are thought to influence the occurrence and severity of leaky gut, depending on the combination and content of the components. The influence of feed components on tail biting and SINS is addressed in the following sections.

### 4.1. Wheat

Wheat and its by-products are often used as the main component in pig feed [151,152]. The protein fraction of wheat has an impact on intestinal integrity in humans. Gluten accounts for 85–90% of the protein in wheat. It is characterized by its specific proteins glutenin and gliadin. Gliadin has peptide sequences (epitopes) that are highly resistant to gastric, pancreatic, and intestinal proteolytic digestion. Various sequences of α, γ, and ω gliadins as well as glutenin induce intestinal disorders [137]. The activation of T cells and increased secretion of IL-6 causes intestinal inflammation. Increased permeability of the epithelium promotes the passage of LPS, MAMPs, and microorganisms [137,138]. Other toxic non-gluten components include amylase-trypsin inhibitors (ATIs), carbohydrate-binding lectin, and rapidly fermentable carbohydrates. ATIs account 2–4% of the total protein in modern wheat. They are albumin proteins that act as plant defense proteins. Some ATIs lead to the release of pro-inflammatory cytokines by monocytes, macrophages, and dendritic cells via the activation of TLR4 [137,139]. The effects of glutenin and gliadin fractions, ATIs, and lectin have been described in great detail in humans [153,154,155]. Comparable studies have not yet been conducted in pigs. However, due to its similar intestinal morphology to humans, the pig is used as an animal model for research in human nutrition [156,157,158]. We hypothesize that similar effects of glutenin and gliadin fractions, ATIs, and lectin can be expected in pigs. Further studies are needed to investigate this hypothesis. If similar effects of wheat could be identified in pigs, this would be a possible cause of tail biting and SINS due to the widespread use of wheat as a feed component. To date, the direct effects of wheat on both conditions have not been investigated in pigs.

### 4.2. Barley

Barley and its by-products are used as a grain component in animal feed [159]. In pig feeding, positive effects on gut health in weaned pigs have been reported. Weaning diarrhea was reduced [160]. In a comparative study, Weiss et al. [140] demonstrated significantly different effects of barley and wheat on ileal and fecal microbiota. In barley-fed pigs Bacteroides, *Clostridium* cluster IV and *Roseburia* spp. were reduced, while *Lactobacillus* spp. was increased in the ileal digesta. *Enterobacteriaceae* and *Bifidobacterium* spp. were reduced in feces of barley-fed pigs. The authors conclude that barley increases the *Lactobacillus* spp./*Enterobacteriaceae* ratio and thus has a beneficial effect on the gut microbiota [140]. Other effects on intestinal health have been observed due to the β-glucans contained in barley. In a study weaned pigs fed with high β-glucan content percentage of CD45RA positive cells in peripheral blood lymphocytes, Peyer’s patches, and mesenteric lymph nodes were increased. Additionally intestinal permeability and *Escherichia* (*E.*) *coli*-enterocyte binding were increased in proportion to β-glucan content in the diet. In conclusion, glucan potentially alters immune and intestinal function in pigs during the weaning period [141]. Arabinoxylans also have an effect on gut health. In a mouse model arabinoxylane promotes the secretion of glucagon-like protein 1 (GLP-1) in enteroendocrine cells and increases the production of SCFAs [144]. Based on beneficial effects of barley on gut health preventive effects on tail biting and SINS could be assumed. However, no studies are available to date that have investigated the effects of barley on the occurrence of tail biting or SINS.

### 4.3. Oats

Oat grain is mainly used in animal feeding and for human consumption [161,162]. The prolamin fraction includes the protein avenin. It has structural similarities to wheat gliadin. Two epitopes have been identified in connection with T cell activation in humans with celiac disease [151,152]. Besides barley, oats contain the highest β-glucan content at 3–8% [162]. In growing pigs, oat β-glucan increases the water binding capacity in small intestine and increases the viscosity of digesta in the colon [153]. In addition, intestinal microbial fermentation of β-glucan leads to increased SCFA formation [154]. Further influences of oats on the gut microbiota have also been identified. In a study with growing pigs, feeding oat bran resulted in an increased abundance of *Prevotella* spp., *Butyricicoccus* spp., and *Catenibacterium* spp. in the hindgut. Additionally, oat bran decreases the expression of IL-8 in the caecum and reduced IL-8, NF-κB, and TNF-α gene levels in the colon [155]. Oats have anti-inflammatory and gut-stabilizing properties that could potentially reduce tail biting and SINS. In addition, the increased viscosity of the digesta would prevent coprostasis, which is a risk factor for SINS [9]. However, such effects of oats on tail biting and SINS have not been studied. Only practical experience suggests possible connections.

### 4.4. Maize

Maize and its by-products are used as main component in pig nutrition [163]. The digestibility of corn starch depends on its amylose content. In a study using a diet containing 40% amylose, Wellington et al. [144] were able to demonstrate effects on the cecal microbiota and fatty acids in weaned pigs. *Lactobacillus* spp. and *Terrisporobacter* spp. were increased and *Streptococcus* spp. were decreased in the caecum. Cecal butyrate and total volatile fatty acids were increased [144]. Further effects are described for different amylose/amylopectin ratios (DARs). Weaners fed with a DAR of 0.40 showed higher diarrhea rates. Amylose supplementation promotes intestinal health. In addition to effects on the intestinal microbiota, cecal acetic and propionic acid were increased and cecal crypt depth is reduced during feed transition after weaning. Under LPS stress, claudin expression in the ileum was increased and the concentrations of the cecal isobutyric and isovaleric acids were increased [145]. The insoluble fibers in maize also have an effect on gut health. Liu et al. [146] were able to demonstrate positive effects on anti-inflammatory response and the diversity of the intestinal microbiota by feeding weaned pigs a diet with 5% corn bran. In blood plasma, the anti-inflammatory cytokine IL-10 was significantly increased. The phylum Firmicutes was decreased while the phylum Bacteroidetes was increased. In particular, *Eubacterium corprostanoligenes*, *Pevotella* spp., and *Fibrobacter* spp. were increased [146]. The amylose contained in maize has the potential to reduce tail biting and SINS. The increased stability of the intestinal barrier under LPS [145] would combat SINS at its origin [8]. Anti-inflammatory effects and beneficial impacts on the gut microbiota could also have a reducing effect on both clinical conditions. Causal links between maize feeding and tail biting as well as SINS have not yet been established.

### 4.5. Soybean Meal

Soybean meal, a by-product of soybean oil production, is used as a main component and major source of plant protein in pig feed [164]. However, macromolecules, anti-nutritional factors (ANFs), and allergens cause hypersensitivity in piglets. Approximately 30% of the total protein in soybean meal is antigenic and is composed mainly of β-conglycinin and glycinin. Protein-based ANFs like trypsin inhibitor inhibits pancreatic protease, proteolysis and protein absorption [147,148,164]. In a study of Wang et al. [149] with weaned piglets β-conglycinin and glycinin induce intestinal endoplasmic reticulum-stress, reduce the expression of tight junctions and Mucin 2 as well as induce epithelial cell apoptosis in the gut. Furthermore, the microbiota phyla and genera levels are affected. The phyla Firmicutes and Actinobacteriota increase, while Bacteroidota decrease. At the same time, the *Lactobacillus* and *Prevotella* genera are reduced [149]. Various processes can be used to inactivate ANFs. Trypsin inhibitors can be inactivated by different heating methods [165,166] or fermentation [167] to make the soybean meal safe for use as feed component.

Soybean meal contains high levels of protein as well as high levels of isoflavones. Isoflavone mainly includes glucosides such as daidzin, genistin, malonyldaidzin and malnylgenistin. Beneficial effects on the small intestine have been observed [150]. In a study on weaned pigs, Li et al. [150] identify increased expression of tight junctions in the jejunum. Particularly, the effects of isoflavones in soybean meal could potentially reduce tail biting and SINS through increased expression of tight junctions. The effects of soybean meal on the occurrence of SINS and tail biting have not yet been investigated. However, a study on influence of a protein-reduced diet on the severity of tail biting has been performed (Table 1) [55].

## 5. Feeding of Undocked Pigs

The systemic inflammation associated with leaky gut and the presumed resulting risks of SINS and tail biting behavior give particular importance to feeding [23,80,133,168,169] and water supply [81,170,171,172] for maintaining gut health.

### 5.1. Importance of Feed Composition for Gut Health

Feeding has a decisive influence on the health and gut health of pigs. Fiber content, protein content, starch content, amino acid content, and plant secondary metabolites are named as factors in the diet [173]. Furthermore, the effects of different nutrient components and feed processing on gut health are described and examined in relation to tail biting and SINS.

#### 5.1.1. Crude Fiber

Dietary fiber is important for physiological gut function. However, high fiber content has a negative effect on feed intake and the energy value of the diet in monogastric animals. By definition, non-starch polysaccharides (NSPs) and lignin belong to the fraction of digestible fibers in feed and can be used to measure fiber content [174]. The digestibility of dietary fiber varies between 40% and 60% and is lower than that of other nutrients like starch, sugars, fat and crude protein. Gut health benefits most from diets containing soluble NSP. Soluble dietary fiber includes pectins, β-glucans and hemicelluloses. However, cellulose and lignin compromise the soluble fiber [175]. Yang et al. [176] describe increased formation of SCFA by the microbiota in the large intestine as an important factor. Microbial fermentation lowers the pH in the gut, and SCFA stimulates the proliferation of epithelial cells, modifies gene expression for epidermal growth factor, and repairs damaged epithelial cells [176]. Liu et al. [23] were able to demonstrate positive effects of dietary fiber in sows in mid- and late gestation, as well as on their offspring. Systemic inflammation in the sows’ intestines was reduced. At the same time, the piglets born showed fewer developmental disorders such as IUGR (intrauterine growth retardation) and increased growth performance. Beneficial bacteria (e.g., *Prevotellaceae*, *Lachnospiraceae*) were increasingly detected in the gut of sows. The authors propose that proliferation of beneficial bacteria reduces the quantity of endotoxins. This reduces inflammation in the gut and increases the integrity of the intestinal barrier [23]. The effects of crude fiber on intestinal integrity, systemic inflammation, and reduced levels of endotoxins in the gut allow conclusions to be drawn about the mechanism of roughage and support our hypothesis (Figure 2). Similar effects were observed in tail biting [56,61] and SINS [9] when fiber sources were fed.

#### 5.1.2. Crude Protein

Dietary protein is fermented by gut microbiota. High-protein diets promote the fermentation of dietary protein in the large intestine. This produces various metabolites such as branched-chain fatty acids (BCFAs), ammonia, amines, phenols, and indoles [177]. These bacterial metabolites can affect the intestinal epithelium. Water absorption, electrolyte secretion and absorption, epithelial barrier function, and colonic epithelial renewal are all influenced [178]. Richter et al. [179] describe reduced expression of the tight junction proteins claudin-1, -2, and -3 in the colon of pigs when there are high proportions of fermentable crude protein in the diet. Diarrhea caused by the fermentation of dietary proteins occurs frequently in pigs after weaning [179]. The fermentation of protein is often linked to the growth of potential pathogenic bacteria such as β-haemolytic enterotoxigenic strains of *E. coli* (ETEC). Another factor is the change in epithelial morphology with a decrease in villus height [177,180]. Effects of protein levels have been observed in tail biting [55]. No studies are yet available on SINS that examine protein levels and the severity of SINS. However, it can be assumed that crude protein can trigger SINS due to its influence on intestinal integrity [8].

#### 5.1.3. Starch

Starch is made of the two polymers, amylose and amylopectin, and can be classified according to its digestibility as rapidly digestible, slowly digestible, and resistant starch [181]. After weaning, starch digestion of the small intestine is disrupted due to insufficient pancreatic enzyme production. If piglets are weaned at 3 weeks of age, this phase can last up to 10 days. The large amount of starch that enters the colon is fermented by bacteria, resulting in increased concentrations of potentially toxic metabolites such as ammonia, phenols, indoles, and secondary bile acids is higher in the distal colon than in the proximal colon [182]. Gao et al. [181] show that weaned piglets fed diets high in amylose had higher villus height in the jejunum and ileum. In addition, the total percentage of apoptotic cells was significantly decreased in these piglets. Gene expression of B-cell lymphoma-2 and occludin in the duodenum and jejunum was increased. In the apical intercellular region of the jejunal epithelium, there was an increase in zonula occludens 1 (ZO-1) protein localization. The authors conclude that gut health and barrier integrity are improved by high amylose levels in young piglets [181]. Resistant starch has positive effects on intestinal health. Bacterial fermentation in the hindgut increases formation of SCFAs, which have beneficial effects on intestinal epithelium (reviewed in [183]) and the intestinal microbiota [184]. The effects of amylose on intestinal morphology and the intestinal barrier can probably counteract the development of SINS. To date, no studies are available that investigate the effects of starch components on SINS and tail biting. According to our hypothesis, amylose would prevent leaky gut and reduce tail biting and SINS.

#### 5.1.4. Amino Acids

Dietary amino acids (AAs) influence gut health and functions in pigs. Various essential and non-essential AAs such as lysine (Lys), tryptophan (Trp), methionine (Met), and threonine as well as arginine (Arg), glycine, cysteine, glutamate (Glu), and glutamine (Gln) have an effect on gut health [185,186]. Positive effects of Lys on the microbiota of the small intestine were demonstrated in piglets during the rearing phase. The *Lactobacillaceae* family occurred significantly more frequently in the small intestine [187]. Trp influences the diversity of microbiota in the large intestine of weaners. In addition, intestinal mucosal IL-8 mRNA levels were reduced [188]. Tight junction proteins ZO-1 and occludin were upregulated in the colon of weaners [188] and finishers [189]. Met reduces oxidative stress and increases the expression of occludin in the jejunum and ileum of weaners [190,191]. In weaners, significantly greater villus heights and a significant increase in vascular endothelial growth factor levels were observed with increased Arg supplementation [192]. Comparable effects were observed in weaners under LPS challenge. Arg significantly increased the number of immunoglobulin A (IgA)-secreting cells, CD8^+^ (CD, cluster of differentiation) and CD4^+^ T cells in the ileum. At the same time, Arg led to a significant reduction in lymphocyte apoptosis in Peyer’s patches [193]. Yin et al. [194] observed increased expression of jejunal SLC7A7 (solute carrier family 7 member 7) and ileal SLC7A1 (solute carrier family 7 member 1) in mycotoxin-exposed growing pigs receiving Arg supplementation. The authors conclude that dietary Arg plays a protective role in the intestines of pigs challenged with mycotoxins [194]. Similar effects were observed with Glu supplementation in growing pigs. Glu improved the imbalance of the antioxidant system and mycotoxin-induced alterations of the intestinal structure [195]. Effects of Gln on the gut barrier were observed in weaned pigs infected with *E. coli*. The expression of the tight junction proteins claudin-1 and occludin remained unchanged in piglets receiving Gln supplementation [196]. Amino acids have various stabilizing and anti-inflammatory effects in the gut. To date, studies on tail biting have been conducted with varying levels of tryptophan [54]. Low tryptophan levels led to increased tail biting. Studies on SINS have demonstrated increased tryptophan metabolism [124]. The effects of amino acids, and tryptophan in particular, clearly support our hypothesis.

#### 5.1.5. Mycotoxins

*Fusarium* spp. produces various mycotoxins found in contaminated feed of pigs [197]. Trichothecenes such as deoxynivalenol (DON) cause erosion of gastric and intestinal mucosa when ingested chronically. Villus atrophy, apical villus necrosis, cytoplasmic vacuolization in enterocytes, edema of the lamina propria, and a significant reduction in goblet cells in the jejunum and ileum have been observed [198]. In addition, the permeability of the intestinal epithelium increased and expression of claudin is reduced under DON exposure [85]. An increase in permeability due to effects on the expression of tight junctions as well as increased interleukins IL-1β and IL-10 already occurs at DON levels that comply with European limits (0.9 mg/kg feed) [199]. The increase in permeability caused by DON leads to paracellular translocation of pathogenic and commensal bacteria from the intestinal lumen [200]. Damage to the intestinal barrier caused by zearalenone (ZEN) has not yet been described. However, influences on the protective mechanisms of the mucosa are evident in the form of increased secretory IgA in the colon [201]. Mycotoxins can affect the intestinal microbiota either directly through antimicrobial action or indirectly through damage to enterocytes [202]. Proteobacteria, and *Lactobacillus* spp. in particular, are negatively affected by DON in the duodenum, jejunum, and ileum [203]. An increased occurrence of *Lactobacillus* spp. has been detected in the colon under the influence of DON and ZEN, which is associated with possible detoxification of both mycotoxins [204]. The permeability-enhancing and pro-inflammatory effects of DON in the intestine promote the occurrence of systemic inflammation, as described as a cause in the pathogenesis of SINS [8,205]. Although no direct effects of mycotoxins on tail biting behavior have been described to date, DON-associated damage to the intestine and the development of systemic inflammation could be an important cofactor in the development of tail biting. Even low DON levels lead to an increase in permeability [199], so DON may not have been considered as a cause. This aspect supports our hypothesis.

#### 5.1.6. Structure of Feed

The size of feed particles has various effects on gastric and intestinal health of pigs. A fine grind improves the digestibility of the feed but increases the risk of gastric ulcers and the growth of enteropathogenic bacteria [206]. Cappai et al. [207] defined the following three classes of ulcerogenic risk of diets for pigs based on the particle size: class 1, high risk (>36% of particles smaller than 400 µm); class 2, moderate risk (29–36% of particles smaller than 400 µm); and class 3, low risk (<29% of particles smaller than 400 µm) [207]. Gastric ulcers are a risk factor for behavioral changes such as loss of appetite and tail biting [208,209,210,211].

High proportions of fine particles accelerate gastric passage of feed. The pH in the feed does not decrease sufficiently [195]. This promotes microbial activity, such as increased proliferation of *Salmonella* spp. and Gram-negative bacteria in the gut [212,213,214]. In the pathogenesis of SINS, increased proliferation of Gram-negative bacteria with an elevation in endotoxin levels in the gut is crucial for the development of systemic inflammation [8,9]. Based on our hypothesis, the degree of grinding would be a factor that promotes the emergence of SINS and tail biting in pigs.

#### 5.1.7. Acid Binding Capacity

In monogastric animals, acid binding capacity (ABC) describes the effects of different feed components and diets on the pH of the stomach [215]. Some feed stuffs bind protons, while other components or additives, such as organic acids, give protons. The ABC is measured as the amount of acid (hydrochloric acid, HCl) in milliequivalents (mEq) required to lower the pH of 1 kg sample material to pH 4.0 (ABC-4) or pH 3.0 (ABC-3) [215,216]. For cereals and vegetable protein, the average values given for ABC-4 and ABC-3 are 72 mEq/kg and 180 mEq/kg, and 14 mEq/kg and 276 mEq/kg, respectively [217]. Stas et al. [218] suggest potential options for creating a diet with low ABC-4 based on the ABC of individual feed components. In general, diets with an ABC above 700 mEq/kg are associated with a high incidence of post-weaning diarrhea in pigs [215]. This implies that high bacterial loads accumulate in the intestine if ABC is insufficient [218], even in combination with a fine degree of grinding [212,213,214]. Intestinal pathogens increase the permeability of the intestinal barrier and increase the risk of a leaky gut [82]. ABC can therefore be considered a risk factor in line with our hypothesis.

### 5.2. Importance of Water Supply for Gut Health

Although water supply is routinely listed among the basic husbandry requirements for pigs, accumulating evidence indicates that it remains an underappreciated driver of tail biting and SINS in pigs (Table 3).

An impaired access to drinking water is identified as a direct precursor of competition, frustration, and redirected oral manipulation toward pen mates. However, the factor water supply is frequently ranked below feed, enrichment, and climate in scientific prioritizations [3,4]. Controlled trials in growing pigs demonstrate that drinker design and flow rate affect both performance and abnormal behavior. Torrey et al. [222] found that bowel drinkers increased water intake and reduced belly nosing compared with nipples, while more recent work by Vande Pol et al. [225] and Scaff [226] quantified a strong linear relationship between water disappearance, body weight, and feed intake. For finisher pigs, Larsen and Pedersen [223] showed that drinker use depends on drinker location, stocking density, and tail damage events. During tail biting outbreaks, the frequency of water use was increased. In addition, the standard pig-to-drinker ratios can still result in restricted access of water in high-density pens [223]. The water requirement of pigs increases steadily with age and performance stage as well as by environmental conditions [227]. Under heat stress, the water requirement for growing and fattening pigs increases by 1.45% with every degree of temperature increase above an ambient temperature of 22 °C [228]. Insufficient water intake combined with heat stress and poor feed characteristics slows down the transport of feces in the intestine and affects gut health [229]. In a study on different pig farms, an improved water comfort via bowel drinkers increased water intake and were significantly associated with fewer necrotic tail lesions and lower tail biting behavior in growing pigs [230]. Recent work on SINS demonstrate that reduced water intake of gestating sows and associated constipation were linked with higher SINS scores and increased early piglet losses. The authors conclude that SINS in early life was associated with later tail damage at slaughter [9,115,117]. This highlights the importance of water supply for gut health and the occurrence of leaky gut as well as systemic inflammation at all ages.

Water quality also plays a crucial role in the gut health of pigs. Microbial biofilms in water pipes affect water quality regardless of whether public water supply or well water is used on the farm [231]. Latent contamination with endotoxins can occur in water [82]. In humans, the effects of water quality on the intestinal microbiome have been described. Colonization of microbial organisms in the intestine [232] and effects on inflammatory bowel diseases occur [233]. The use of water disinfection improves water quality and has a positive effect on the gut microbiome. In fattening broilers, chlorination of the water promoted the occurrence of beneficial microorganisms and increased fattening performance [234]. According to our hypothesis, water supply and water quality are important factors for the development of leaky gut-associated systemic inflammation.

### 5.3. Potential of Feed Ration to Prevent Systemic Inflammations and Tail Biting

Based on the described properties of the ingredients and nutritional components and their effects on gut health, it can be suggested that a gut-friendly diet can be formulated which may reduce the risk of tail biting and SINS in pigs. Our previous work describes various diets for growing pigs and finishers on a conventional farm with undocked pigs, which were prepared and optimized on the basis of a SINS scoring system [235] (Table 4).

The presented diets (Table 4) for growing pigs and finishers with body weight (BW) > 40 kg initially contain high proportions of barley (59%, 50%, and 41%) and low proportions of wheat (11%, 23%, and 20%) as cereal components. At BWs of >40 kg during finisher stage barley (41%), wheat (20%), and triticale (20%) are fed in similar proportions. Soy is used as a protein source in all stages. For growing pigs with BWs > 10 kg and >20 kg, the soybean meal is fed at levels of 15% and 17%, respectively. When these diets (Table 4) are compared with other rations from trials with undocked pigs (Table 1), differences in the wheat content of the rations during growing stage are noticeable. Diets with wheat contents between 30% and 59% show prevalences of tail biting up to 70% in weaners [56,59,60] (Table 1). Chou et al. [57] found a comparatively low prevalence of tail biting in weaners with 13.69–22.32% using equal proportions of wheat and barley (Table 1).

When comparing the nutritional components of diets for growing pigs in Table 4 with diets for the same age group from feeding trials with undocked pigs (Table 1), differences in crude protein and crude fiber content are noticeable. In the studies of Kauselmann et al. [58,59,60,61], crude protein contents of 16.1% and 17.8% were fed during rearing weeks 1–2 and 3–8. The prevalence of tail biting varied between 15.0% and 42.2% across studies [58,59,60,61]. In the diets for growing pigs in Table 4, crude protein contents of 15% and 16% were used during the same rearing periods, which were lower than the contents used in the studies of Kauselmann et al. [58,59,60,61]. The situation is similar with regard to the crude fiber content in growing piglets. The crude fiber contents in Table 4 at 4.7% and 4.4% are above the crude fiber contents of 4.4% and 4.1% used for rearing pigs of the same age in Table 1 [58,59,60,61]. The diets in Table 4 contain with 0.2% higher tryptophan levels than those in the study by Roux et al. [56], which contained 0.19%. In the study, the prevalence of tail biting was 30–70%.

In summary, positive effects can be concluded from low wheat content combined with high barley content, as well as higher crude fiber content combined with lower crude protein content during the rearing phase. It can also be assumed that an increased proportion of tryptophan at 0.2% in all age groups has a reducing effect on tail biting and SINS. These observations are consistent with our hypothesis and have the potential to achieve a reduction in tail biting and SINS.

## 6. Preparation of Feed Ration Based on Behavior and Inflammatory Lesions

In previous studies, little importance was attached to the influence of feeding as a cause of tail biting. The focus of research continues to be on the effects of enrichment materials and housing conditions [6,55,56,57,58,59,60,61]. However, the available literature indicates that gut health has an influence on tail biting [94,99,100,101,102,103,104,105,106,107,108,112] and SINS [8,9,41,114,115,116,117]. Based on the literature and various published research findings, our hypothesis of leaky gut-associated systemic inflammation as a cause of tail biting and SINS in pigs is derived (Figure 2).

### 6.1. Prevention of Tail Biting and SINS by Improving Gut Health and Feed Composition

Various external and husbandry-related factors influence gut health and promote the occurrence of leaky gut. Feeding [23,80], water supply [81,82], heat stress [83], infections [84], mycotoxins [85] and social stress [86] negatively affect gut health and the integrity of the intestinal barrier (Figure 1). Feeding in particular plays a decisive role in the development of leaky gut. Initial success in preventing systemic inflammation and tail biting in pigs has been achieved through a diet composition based on a SINS scoring system [235]. This allows us to derive possible measures for improving gut health and ration design. Feed composition through SINS scoring and tail biting scoring could be one way to improve gut and animal health. Koenders-van Gog et al. [115] describe SINS scores as an indicator of animal health and welfare. The authors characterize the gut as an important immune organ and early SINS symptoms as an inflammatory response that indicates the competence of the innate and adaptive immune system of pigs [115].

### 6.2. Scoring of Tail Biting and SINS

Scoring systems include various organs of pigs in all stages have already been described for SINS scoring [9,12,41,114,115]. Various scoring systems are described for tail biting in pigs [236,237]. In general, tail biting is scored based on behavior [1,238,239], tail posture [36,37] tail lesions [236,237], vocalization [240], and tail injuries on carcasses [237,241,242]. With the help of these established scoring systems, the prevalence of tail biting and SINS in the herd can be determined.

### 6.3. Examination of External Risk Factors

The next step should be to examine external factors that increase the risk of tail biting [3,4,5,6], SINS [9,10,11], and the occurrence of leaky gut [3,80,81,82,83,84,85,86]. European legislation (Council Directive 2008/120/EC and European Recommendation 336/2016) already states that risk factors leading to tail biting in pigs must be recorded. Various scoring systems associated with tail biting are described in the literature for the targeted assessment of husbandry factors [243,244,245,246].

### 6.4. Adaptive Feedback Mechanism for Evaluating Effective Measures

**Figure 3:**
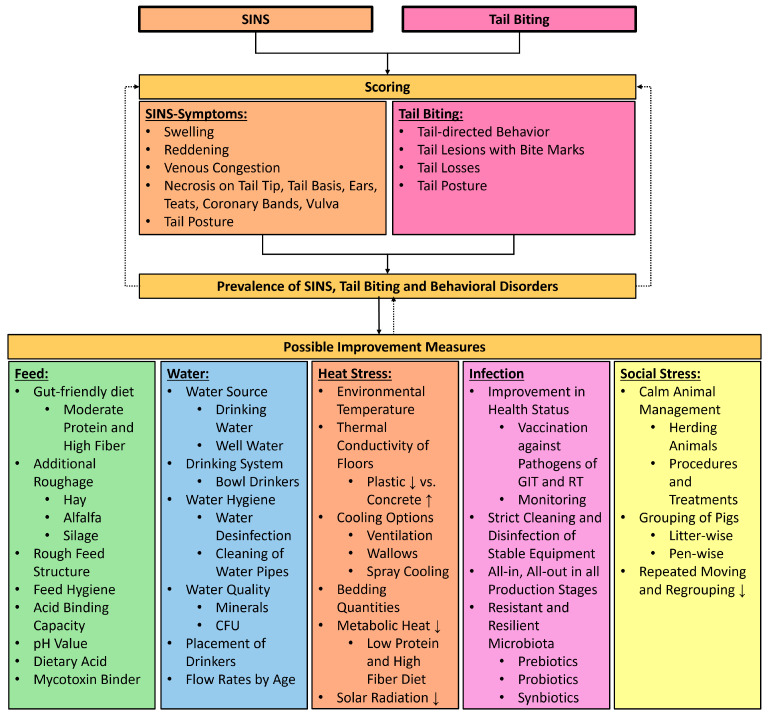
Conceptual model of a measure and feedback cycle for optimizing feeding and husbandry-related factors based on SINS and tail biting prevalence. The effects of a diet can be assessed by the occurrence of SINS and tail biting. Due to various influences on gut health, these factors must also be improved in order to achieve health and integrity of undocked pigs through a stable intestinal barrier. CFUs, colony forming units; GIT, gastrointestinal tract; RT, respiratory tract; dotted arrows, Feedback mechanism for assessing symptom prevalence and implemented measures; ↑, increase; ↓, decrease.

Based on our hypothesis and a diet created for the first time using SINS scoring, we conclude that a combination of optimizations in husbandry-related factors with SINS scoring and behavioral scoring can improve the feeding and management of undocked pigs. In an adaptive feedback mechanism, the effects of feeding and measures can be examined, assessed, and modified as necessary using SINS scoring and behavioral scoring (Figure 3).

### 6.5. Potential Economic Impact of Preventive Measures

Implementing measures to prevent tail biting and SINS is costly for commercial farms. However, tail biting is an important economic factor. Even at prevalence levels of 10%, this can reduce the income per fattening pig by up to €18.96 [24,25]. Preventive measures to reduce tail biting are effective in reducing economic losses. However, their success must be assessed on a farm-by-farm basis, as various factors such as farm structure, initial conditions on the farm, sustainability in the implementation of measures, and the health status of the herd are decisive [25]. The cost–benefit factor of the optimization measures in Figure 3 cannot be fully estimated due to insufficient data available in the literature. In addition, there are possible variables such as legal regulations, national support programs, and marketing channels that could influence actual investments and running costs. Based on the results of Niemi et al. [247], a positive effect on revenue can be expected from investments in the mentioned optimizations. Comparable data on economic losses caused by SINS have not yet been published. Further studies are necessary to determine the exact effects of optimizations to prevent tail biting and SINS on the economic profitability of the farm.

## 7. Conclusions

In previous studies with undocked pigs, the composition of the feed had negligible relevance to the development of tail biting. The focus was primarily on organic enrichment material, which were considered to have nutritional effects. However, numerous studies on feed components used show that beneficial and harmful effects on gut health and the integrity of the intestinal barrier can be induced. Depending on the composition of the diet, the risk of leaky gut and associated systemic inflammation increases. The endogenous development of SINS has been described in this context in the past, and modifications to feeding practices have been implemented for prevention of SINS.

Systemic inflammation also affects the behavior of pigs. Increased activation of IDO by an endotoxin-derived inflammatory response can induce behavioral changes such as tail biting through neurotoxic metabolites. Based on systemic inflammation, a link to SINS is a plausible mechanism for some instances of tail biting in pigs. It is another piece of the puzzle for targeted control strategies to reduce both SINS and tail biting. Stable gut health and an intact intestinal barrier are key factors that must be considered as a requirement for keeping undocked pigs. Further studies are needed to confirm this hypothesis of leaky gut-associated systemic inflammation as common etiology for SINS and tail biting.

A major limiting factor of this work is the lack of knowledge and the available literature addressing causal relationships between gut health and tail biting, as well as feeding strategies to prevent tail biting and SINS. Based on a broad literature review, various effects were identified and leaky gut-associated systemic inflammation was suspected as a possible common cause of tail biting and SINS. Further comparative studies with different diets and feed components are necessary to investigate the effects of feeding and the influence of gut health on the prevalence of both clinical conditions.

## Figures and Tables

**Table 1:** Main components in the feeding of undocked pigs in different studies.

Age	Feed Components (%)		Prevalence of Tail Biting (%)	Key Findings	Reference
Weaners	50.0 5.1 13.6 20.0 0.5 1.9 3.8 2.0 9.8 MJ 12.9 4.1	Barley Oat Maize Wheat Wheat Flour Potato Protein Rapeseed Press Cake Dried Beet Pulp Per kg Dry Matter DE Crude Protein Crude Fiber	14.7 ^a^	No evidence in relationship of breeds with higher protein efficiency and harmful behavior	[55]
Weaners	34.3 25.0 15.0 5.0 5.8 8.0 9.6 MJ 14.4 18.0	Wheat Barley Maize Wheat Bran Soybean Meal Sunflower Meal Per kg Dry Matter DE Crude Protein Crude Fiber	30.0–70.0 ^a^	Diet with 6% fermentable fiber and 0.19% digestible tryptophan increase significantly intact tails (*p* < 0.1)	[56]
Weaners	18.0 25.0 15.36 18.0 16.26 MJ 19.6 3.7	Wheat Feed Flour Soybean Meal (48% Protein) Maize Barley Per kg Dry Matter DE Crude Protein Crude Fiber	13.69 ^b^	Increased fiber content in the diet in combination with one organic (wood) or one non-organic (rubber floor toy) enrichment material had no reducing effect on tail biting	[57]
Weaners	18.0 25.0 15.4 18.0 1.0 15.9 MJ 19.3 5.3	Wheat Feed Flour Soybean Meal (48% Protein) Maize Barley Wheat Bran Per kg Dry Matter DE Crude Protein Crude Fiber	22.32 ^b^	Increased fiber content in the diet in combination with one organic (wood) or one non-organic (rubber floor toy) enrichment material had no reducing effect on tail biting	[57]
Weaners Week 1–2 of Rearing	18.0 20.0 20.0 5.0	Barley Wheat Oat Flakes Maize Flour	15.0–30.0 ^c^	Maize kernels as enrichment material increase exploration and competitive behavior	[57]
	14.1 MJ 16.1 4.4	Per kg Dry Matter DE Crude Protein Crude Fiber	37.7 ^c^	Flavored straw pellets have no significant effect on the occurrence of tail injuries	[58]
Weaners Week 3–8 of Rearing	32.0 40.0 16.0	Barley Wheat Soybean Meal	42.2 ^c^	Frequently filled dispenser with chopped straw affects tail damage	[59]
	13.9 MJ 17.8 4.1	Per kg Dry Matter DE Crude Protein Crude Fiber	15.0–35.0 ^c^	Alfalfa and straw pellets decrease tail length losses	[60]
Finishers	50.0 6.3 16.2 20.0 0.5 0.1 2.4 2.0 9.9 MJ 11.2 4.1	Barley Oat Maize Wheat Wheat Flour Potato Protein Rapeseed Press Cake Dried Beet Pulp Per kg Dry Matter DE Crude Protein Crude Fiber	14.7 ^a^	No evidence in relationship of breeds with higher protein efficiency and harmful behavior	[55]
Finishers	30.2 30.0 16.2 5.6 3.5 8.0 9.6 MJ 13.03 18.3	Wheat Barley Maize Wheat Bran Soybean Meal Sunflower Meal Per kg Dry Matter DE Crude Protein Crude Fiber	30.0–70.0 ^a^	Diet with 6% fermentable fiber and 0.19% digestible tryptophan increase significantly intact tails (*p* < 0.1)	[56]
Finishers	45.4 13.5 6.0 24.0 8.5 14.9 MJ 14.2 5.9	Wheat Feed Flour Soybean Meal (48% Protein) Maize Barley Soybean Hulls Per kg Dry Matter DE Crude Protein Crude Fiber	13.69 ^b^	Increased fiber content in the diet in combination with one organic (wood) or one non-organic (rubber floor toy) enrichment material had no reducing effect on tail biting	[57]
Finishers	47.6 26.3 13.5 5.8 13.7 MJ 15.4 11.6	Wheat Feed Flour Soybean Hulls Soybean Meal (48% Protein) Maize Per kg Dry Matter DE Crude Protein Crude Fiber	22.32 ^b^	Increased fiber content in the diet in combination with one organic (wood) or one non-organic (rubber floor toy) enrichment material had no reducing effect on tail biting	[57]
Finishers Week 1–7 of Fattening	17.0 59.0 19.0	Barley Wheat Soybean Meal	15.0–30.0 ^c^	Maize kernels as enrichment material increase exploration and competitive behavior	[58]
	14.0 MJ 17.06 3.7	Per kg Dry Matter DE Crude Protein Crude Fiber	37.7 ^c^	Flavored straw pellets show a trend toward reducing tail injuries	[59]
Finishers Week 8–11 of Fattening	59.0 22.0 15.0	Barley Wheat Soybean Meal	42.2 ^c^	Frequently filled dispenser with chopped straw affects tail damage	[60]
	13.9 MJ 17.8 4.1	Per kg Dry Matter DE Crude Protein Crude Fiber	15.0–35.0 ^c^	Chopped hay increases exploration, but has no effect on prevalence of tail biting	[61]

^a^ the prevalence of tail biting was not differentiated by ration or age in the cited studies [55,56]. ^b^ the prevalence of tail biting was reported by ration (standard diet; high fiber diet) in the cited study [57]. No differentiation by age was made. ^c^ the prevalence of tail biting was reported by age (weaners; finishers) in the cited studies [58,59,60,61]. No differentiation by ration was made. MJ, megajoule; DE, digestible energy.

**Table 2:** Summarized effects of dietary components on gut health.

Component	Effects on Gut Health		Reference
Wheat	Gliadin, Glutelin	T cell activation ↑ IL-6 ↑ Permeability ↑ Passage of LPS, MAMPs and MOs ↑	[137,138,139]
	ATIs, Lectin	Pro-inflammatory cytokines ↑	
Barley	Ileum	*Lactobacillus* spp. ↑ Bacteroides ↓ *Clostridium* cluster IV ↓ *Roseburia* spp. ↓	[140]
	Colon	*Enterobacteriaceae* ↓ *Bifidobacterium* spp. ↓	
	β-Glucans	CD45RA positive cells ↑ Permeability ↑ *E. coli*-enterocyte binding ↑	[141]
	Arabinoxylans	GLP-1 ↑ SCFA ↑	[142]
Oats	Avenin	T cell activation ↑	[151,152]
	β-Glucans	Small intestine: digesta WBC ↑ Colon: digesta viscosity ↑ SCFA ↑	[153,154]
	Caecum	IL-8 ↓	[143]
	Colon	IL-8 ↓ NF-κB ↓ TNF-α ↓ *Prevotella* spp. ↑ *Butyricicoccus* spp. ↑ *Catenibacterium* spp. ↑	[143]
Maize	Amylose	*Lactobacillus* spp. ↑ *Terrisporobacter* spp. ↑ *Streptococcus* spp. ↓ Caecum: crypt depth ↓ Acetate ↑ Butyrate ↑ Propionate ↑ Total VFA ↑	[144,145]
	Insoluble Fiber	Firmicutes ↓ Bacteroidetes ↑ *Eubacterium corprostanoligenes* ↑ *Pevotella* spp. ↑ *Fibrobacter* spp. ↑	[146]
Soybean Meal	β-Conglycinin, Glycinin	Tight junctions ↓ Mucin 2 ↓ Epithelial cell apoptosis ↑ Intestinal ER-Stress ↑ Firmicutes ↑ Bacteroidota ↓ Actinobacteriota ↑ *Lactobacillus* spp. ↓ *Prevotella* spp. ↓	[147,148,149]
	Isoflavone	Tight junctions ↑	[150]

ATIs, amylase-trypsin inhibitors; *E. coli*, *Escherichia coli*; ER, endoplasmic reticulum; GLP-1, glucagon-like protein 1; IL, interleukin; MAMPs, microbial-associated molecular patterns; MOs, microorganisms; LPS, lipopolysaccharide; NF-κB, nuclear factor kappa-light-chain-enhancer of activated B-cells; TNF-α, tumor necrosis factor alpha; VFA, volatile fatty acids; WBC, water binding capacity; ↑, increase; ↓, decrease.

**Table 3:** Summarized effects of water supply on tail biting and SINS in pigs.

Water Supply	Effects		Reference
Nipple Drinkers	Low Flowrate	Drinking Time ↑ Competition for Water ↑	[219]
		Tail Biting ↑	[220,221]
	Normal Flowrate	SINS ↑ Coprostasis in Sows ↑	[9,115]
		Inflammatory Metabolism ↑	[114,124]
	Higher Flowrate	Drinking Time ↓ Water Intake ↑ Growth Performance ↑	[219]
		Tail Biting ↓	[220,221]
Bowel Drinkers	Higher Flowrate	Water Intake ↑ Abnormal Pre-biting Behavior ↓	[222]
	Open Water Source	SINS ↓ Coprostasis in Sows ↓	[9]
		Inflammatory Metabolism ↓	[124]
	Water Disinfection	SINS ↓	[9]
Drinker Location	Near Feeder	Water Intake ↑	[223]
	Opposite Feeder	Water Intake ↓	[223]
Drinking Frequency	Tail Damage Event	Frequency ↑	[223]
	Higher Pig to Drinker Ratio (≥1:12)		[224]
	Intact Tail	Frequency ↓	[223]
	Low Pig to Drinker Ratio (<1:12)		[224]

SINS, swine inflammation and necrosis syndrome; ↑, increase; ↓, decrease.

**Table 4:** Main components of different diets for weaners and finishing pigs, prepared and optimized using a SINS scoring system, on a German farm with undocked pigs [235].

Ingredients	Growing Pigs ^1^ BW > 10 kg	Growing Pigs ^1^ BW > 20 kg	Finishers ^2^ BW > 40 kg	Finishers ^2^ BW > 60 kg	Finishers ^2^ BW > 90 kg
Barley (10.4% CP) %	59.0	50.0	41.0	27.0	21.0
Wheat (11.9% CP) %	11.0	23.0	20.0	27.0	30.0
Triticale %	-	-	20.0	27.0	30.0
Soybean Meal (HP 46%) %	15.0	17.0	10.0	8.0	5.0
Nutrient Components (88% DM)					
ME MJ/kg DM	12.0	12.2	12.8	12.9	13.0
Crude Protein %	15.0	16.0	14.9	14.8	14.5
Crude Fiber %	4.7	4.4	4.3	3.9	3.9
Starch %	34.8	34.8	43.2	44.3	44.8
Tryptophan %	0.2	0.2	0.2	0.2	0.2

Conventional closed herd with 220 breeding sows (breed: Topigs TN 70; insemination boar: Danish Duroc), 1700 rearing pigs and 1200 fattening pigs. Rearing and fattening pens with solid concrete bed surface and slatted cast iron grid in covered runs, dry feeding, water supply via bowel drinkers with water disinfection. SINS prevalence < 5%. Tail biting prevalence < 2%. ^1^ per ton of feed addition of 3 kg feed coal. ^2^ per ton of feed addition of 5 kg feed coal. BW, body weight; CP, crude protein; DM, dry matter; HP, high protein; ME, metabolic energy; MJ/kg, megajoule per kilogram.

## Data Availability

No new data were created or analyzed in this study.
